# The West London Hospital and the Three Examining Boards in London

**Published:** 1915-07-31

**Authors:** N. Bishop Harman, Frank Haydon, F. G. Hallett, P. J. Hartog

**Affiliations:** Vice-Dean. The Post-Graduate College, West London Hospital, Hammersmith.; Secretary. Blackfriars, London, E.C.; Secretary. Examination Hall, 8-11 Queen Square, Bloomsbury, London, W.C.; Academic Registrar. South Kensington, London, S.W.


*To the Editor of* The Hospital.

### The West London Hospital and the Three Examining Boards in London.

*To the Editor of* The Hospital.

Sir,—I am directed to forward to you the enclosed copies of correspondence, with a request that you will be pleased to publish them in your columns. The first letter is one addressed to the three examining boards in London; the replies to that letter follow.

The request made by the West London Hospital appears to be reasonable, and of urgent necessity in these days of war emergency. One examining board recognises this emergency and grants the request. The other two refuse it, and, perhaps wisely, state no reason for their refusal.

Emergency has cut much red tape of late; even trade-union rules have gone by the board. But our examining boards still "bind heavy burdens and grievous to be borne, and lay them on men's shoulders, but they themselves will not move them with one of their fingers."—Yours faithfully, N. BISHOP HARMAN, Vice-Dean.

The Post-Graduate College,
West London Hospital, Hammersmith.
July 20, 1915.

[We hope to deal with the matter in our next issue.—ED. THE HOSPITAL.]

---

*Copy of Letter to the Three Examining Boards in London.*

Dear Sir,—Owing to the necessity for releasing medical men of military age, there is increasing difficulty in obtaining the necessary skilled assistance for the satisfactory working of general hospitals. The difficulty has been acutely felt at the West London Hospital. At the present time we are able in some degree to meet it by obtaining the assistance of senior students who are as yet unqualified, who work under the direction of the resident medical officers as dressers for surgical cases. These men are paid for the work, and found in board and lodging. To make this work really suitable for them without delaying the date at which they would ordinarily be able to proceed to their final examinations, I am authorised to make application to you for the official recognition of this hospital as a place of clinical study for men who have passed their intermediate examination. The hospital is at present recognised as a place of study for post-graduates, and for men reading for higher professional qualifications. There is ample clinical material of all kinds, and full facility for personal teaching by the staff, both resident and visiting. We have at the moment two men acting as dressers in the surgical wards, and I shall be glad if your council will be pleased to grant the request made in this letter—that you will make it retrospective so as to apply to these two men. The application for this recognition is for the duration of the war only.—I am, yours faithfully,

(Signed) N. BISHOP HARMAN, Vice-Dean.

The Post-Graduate College,
West London Hospital, Hammersmith.
June 19, 1915.

---

*Copy of Replies from the Three Examining Boards to Mr. N. Bishop Harman, West London Hospital.*

(1) SOCIETY OF APOTHECARIES OF LONDON.

Dear Sir,—I am requested to inform you that the Court of Examiners are willing to recognise work done at the West London Hospital for clinical teaching during the war "for students who have passed their examination in anatomy and physiology."

Would you be good enough to send me the names of the two students who are acting as dressers?—Yours faithfully, (Signed) FRANK HAYDON, Secretary.

Blackfriars, London, E.C.
June 24, 1915.

---

(2) THE CONJOINT BOARD.

Dear Sir,—I have submitted your letter of the 19th instant to the committee of management of this board, and I am desired to inform you that they do not see their way to comply with your request.—I am, dear Sir, yours faithfully, (Signed) F. G. HALLETT, Secretary.

Examination Hall, 8-11 Queen Square,
Bloomsbury, London, W.C.
June 24, 1915.

(3) UNIVERSITY OF LONDON.

Dear Sir,—I regret to inform you that, after consideration of your letter of June 19 last, making application for the temporary recognition of the West London Hospital for clinical study for the purposes of the M.B., B.S. examination, and of reports thereon from the relevant committees of the Senate, it was resolved by the Senate at their meeting on July 14, 1915, that the application be not acceded to.—I am, dear Sir, yours faithfully,

(Signed) P. J. HARTOG, Academic Registrar.

South Kensington, London, S.W.
July 15, 1915.

